# Eccentric cycling is more efficient in reducing fat mass than concentric cycling in adolescents with obesity

**DOI:** 10.1111/sms.13301

**Published:** 2018-10-04

**Authors:** Valérie Julian, David Thivel, Maud Miguet, Bruno Pereira, Frédéric Costes, Emmanuel Coudeyre, Martine Duclos, Ruddy Richard

**Affiliations:** ^1^ Department of Sport Medicine and Functional Explorations, Diet and Musculoskeletal Health Team, CRNH, INRA University Teaching Hospital of Clermont‐Ferrand, University of Clermont Auvergne Clermont‐Ferrand France; ^2^ Laboratory AME2P University of Clermont Auvergne Aubière France; ^3^ Department of Biostatistics University Teaching Hospital of Clermont‐Ferrand Clermont‐Ferrand France; ^4^ Department of Physical Medicine and Rehabilitation, Diet and Musculoskeletal Health Team, CRNH, INRA University Teaching Hospital of Clermont‐Ferrand, University of Clermont Auvergne Clermont‐Ferrand France

**Keywords:** childhood obesity, eccentric exercise, body composition, exercise physiology

## Abstract

The benefits of eccentric (ECC) training on fat mass (FM) remain underexplored. We hypothesized that in obese adolescents, ECC cycling training is more efficient for decreasing whole‐body FM percentage compared to concentric (CON) performed at the same oxygen consumption (VO_2_). Twenty‐four adolescents aged 13.4 ± 1.3 years (BMI > 90th percentile) were randomized to ECC or CON. They performed three cyclo‐ergometer sessions per week (30 min per session) for 12 weeks: two habituation, 5 at 50% VO_2peak_, and 5 at 70% VO_2peak_. Anthropometric measurements, body composition, maximal incremental CON tests, strength tests, and blood samples were assessed pre‐ and post‐training. Whole‐body FM percentage decreased significantly after compared to pretraining in both groups, though to a larger extent in the ECC group (ECC: −10% vs CON: −4.2%, *P* < 0.05). Whole‐body lean mass (LM) percentage increased significantly in both groups after compared to pretraining, with a greater increase in the ECC group (ECC: 3.8% vs CON: 1.5%, *P* <0.05). The improvements in leg FM and LM percentages were greater in the ECC group (−6.5% and 3.0%, *P* = 0.01 and *P* < 0.01). Quadriceps isometric and isokinetic ECC strength increased significantly more in the ECC group (28.3% and 21.3%, *P* < 0.05). Both groups showed similar significant VO_2peak_ improvement (ECC: 15.4% vs CON: 10.3%). The decrease in homeostasis model assessment of insulin resistance index was significant in the ECC group (−19.9%). In conclusion, although both ECC and CON cycling trainings are efficient to decrease FM, ECC induces greater FM reduction, strength gains, and insulin resistance improvements and represents an optimal modality to recommend for obese adolescents.

## 1 INTRODUCTION

Obesity, defined as excessive fat accumulation due to an imbalance between energy intake and energy expenditure, is a serious current public health challenge. Approximately 80% of adolescents with obesity will remain obese through to adulthood.^1^ Although physical activity, associated with dietary strategies, proves to be effective in preventing and treating childhood obesity, children and adolescents with excess body weight typically exhibit several functional limitations that diminish their physical capacities (ie, muscle, respiratory, or cardiac limitations),^1^ thus lowering the expected benefit of their training programs.

So far, anti‐obesity strategies involving physical exercise have mainly used classical exercise modalities, based mostly on predominant concentric muscle contraction.^2^, ^3^ To our knowledge, no study has yet evaluated the impact of eccentric cycling in this population. Daily life activities are performed with a combination of concentric (CON) and eccentric (ECC) skeletal muscle contractions. During CON contractions, muscle generates force by shortening, whereas during ECC contractions, muscle generates force by lengthening (developing tension to either decelerate movement or acting against gravity). Three types of ECC training can be distinguished: (a) plyometric exercises (such as drop jumps, with contractions lasting milliseconds and producing thousands of watts of negative power), (b) classical ECC resistance exercises (protocols consisting of near maximal ECC contractions lasting few seconds, used to lift and lower weights), (c) “continuous moderate load ECC exercises”^4^ (also denoted as resistance exercise via negative eccentric work^5^). Thus, alternative training modalities, such as cycling with special motorized ECC cycle ergometers, downhill walking or running, and stepping exercises, have been characterized as “continuous moderate load ECC exercise”.^4^ One of the physiological characteristics of ECC cycling is that it lowers metabolic demand, compared with CON cycling when performed at the same mechanical power ,^5^, ^6^ due to the combination of a high muscle force with low energy cost (ie*,* low oxygen uptake ratio and $\dot{V}O_{2}$/power). This lower metabolic cost is mainly due to a lower level of extensor and flexor muscle activations and lesser muscle deoxygenation during ECC compared with CON cycling.^6^ Eccentric training is particularly suitable for patients with chronic pathologies, resulting in cardiac, respiratory, or muscular limitations to their exercise capacities.^4^, ^7^ Nevertheless, one possible negative effect of ECC modality is exercise‐induced muscle damage (EIMD).^8^ This causes temporary lesions on plasma membranes, contractile and non‐contractile proteins in response to overstretching,^8^ and induces muscle weakness and delayed‐onset muscle soreness (DOMS), particularly in untrained subjects and chronic disease populations. Nevertheless, the repeated bout effect, which includes neural and structural adaptations, protects against muscle damages from subsequent ECC bouts.^9^, ^10^ Thus, an ECC cycling program can be achieved without undue DOMS provided a progressive ramping protocol is followed.^4^, ^11^

Although the impact of ECC training on lean mass (LM) has been extensively studied, the findings are too varied to clearly affirm the superiority of ECC training.^11^, ^12^, ^13^, ^14^ Recent meta‐analyses demonstrated that when matched for load or work, changes between ECC and CON training were found similar in regard to muscle hypertrophy.^15^, ^16^ Considering fat mass (FM), the impact of ECC training remains poorly studied, without direct comparisons between ECC and CON training. Nevertheless, referring to the studies that compared ECC and traditional trainings, ECC training is more^17^ or at least as effective as traditional training^18^, ^19^ at reducing FM. Although the mechanisms of muscle hypertrophy, such as the increases in protein synthesis or in the recruitment of satellite cells,^20^ have been well studied, the physiological and metabolic mechanisms that could reduce adiposity, such as modifications in postexercise resting energy expenditure and metabolic profile, need more investigation.^21^, ^22^ It has been argued that acute and chronic ECC exercise induces a greater increase in postexercise resting energy expenditure, compared with CON exercise performed at the same mechanical power, related to the ECC‐induced elevation protein turnover (ie, an increase in both muscle protein degradation and synthesis).^8^, ^22^ Moreover, it modifies metabolic substrate use, increasing fat oxidation,^21^, ^22^ and favors a postexercise decrease in blood lipid (which would participate in synthesize new cell membranes of injured muscles).^22^, ^23^ Thus, considering the similar or superior potential effects of ECC training on body composition and its lower metabolic demand, ECC training would be more efficient than CON training given the ratio of energy expenditure to net force or work production.

Several methodological issues could explain the relatively limited number of studies that have compared the effects of ECC and CON exercises: (a) the difficulty in isolating ECC and CON actions during typical everyday movements; (b) the rigorous methodology required to compare ECC and CON exercise in standardized experimental conditions of power output (ie, at the same mechanical power) or oxygen consumption (ie, at the same metabolic rate or oxygen consumption [$\dot{V}O_{2}$] level, with mechanical power 3‐5 times higher during ECC cycling); and (c) specific ECC pedal ergometers have only acquired widespread usage in the last decade. However, to date, only a few studies have focused on ECC training in pediatric populations,^24^, ^25^, ^26^, ^27^ none of which have tested ECC cycling training. To our knowledge, the impact of ECC cycling training on all aspects of total and segmental body composition has not yet been explored in adult or young obese patients.

The aim of the present study was to compare the impact of an ECC cycling program vs CON cycling program on whole‐body FM percentage in a population of obese adolescents. The secondary aims were to explore the impact of ECC vs CON cycling training on anthropometric measurements, quadriceps’ strength, aerobic capacities, lipid blood profile, and insulin resistance. We hypothesized that an ECC cycling program would prove more efficient at reducing FM than CON cycling program performed at the same $\dot{V}O_{2}$.

## 2 METHODS

### 2.1 Subjects

We recruited 24 obese adolescents from the Pediatric Obesity Center (Tza Nou, La Bourboule, France; 12 males and 12 females; Tanner stages 3‐4). All had to meet the following criteria: (a) age: 12‐16 years, (b) body mass index [BMI] > 90th percentile (according to the international cutoff points, (c) undergoing no medication affecting energy metabolism, nor regular tobacco or alcohol use, and (d) presenting no contraindications to exercise. All adolescents and their legal representatives received detailed information sheets, were informed, and signed written consent forms as required by ethical rules. The trial was approved by the relevant ethical committee for adolescents, Comité de Protection des Personnes Est IV, IDRCB 2016‐A00043‐48, and registered on ClinicalTrials.gov as NCT02925572.

### 2.2 Study design

All the adolescents underwent a full medical examination to ensure their ability to complete the study and then 12 weeks of traditional medical care at the Pediatric Obesity Center. The care consisted of dietary counseling without energy restriction and a progressive physical activity program. The duration and intensity of the program's activity sessions increased progressively, using activities such as outdoor walking and team sports, to reach a total of 60 cumulative minutes of moderate intensity per day, as recommended by the World Health Organization. Once complete, the adolescents were randomized, using random size blocks, and assigned to either CON or ECC cycling.

**Figure 1:**
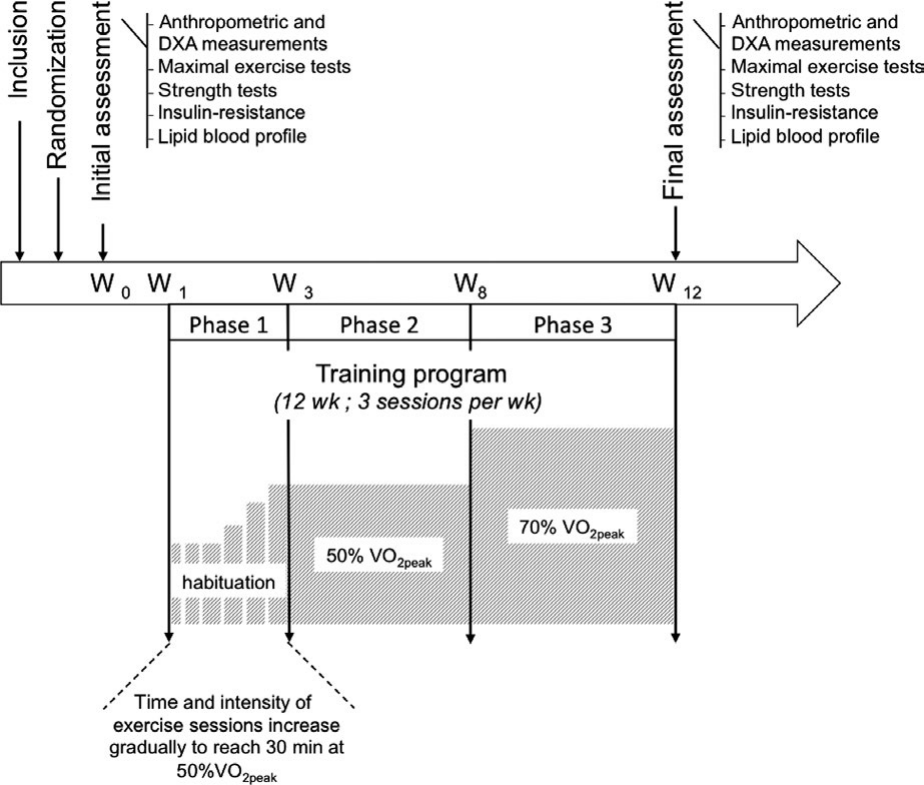
Study design. The training program consisted of 3 cycle‐ergometer sessions/wk during 12 wk: 2 wk of habituation (Phase 1), 5 wk of eccentric (ECC) or concentric (CON) cycling at 50% $\dot{V}O_{2peak}$ (Phase 2), and 5 wk of eccentric (ECC) or concentric (CON) cycling at 70% $\dot{V}O_{2peak}$. Anthropometric measurements, body composition, maximal incremental tests, strength tests, insulin resistance, and blood lipid profile were assessed at pre‐ and post‐training. DXA, dual‐energy X‐ray absorptiometry; $\dot{V}O_{2peak}$, oxygen consumption; W, week

For both training groups, the intervention consisted of a 12‐week cycling program involving 36 ergometer exercise training sessions (3 sessions/wk). Anthropometric measurements, body composition measurements, blood samples, maximal incremental exercise tests, and isometric and isokinetic strength tests were taken before (pretraining session) and after (post‐training session) both interventions. The study design is illustrated in Figure 1.

### 2.3 Food intake

As part of the intervention, all participants received the same nutritional education (45 minutes for every 2 weeks). There was no dietary restriction per se. The adolescents followed a balanced normo‐caloric diet provided and supervised by the Pediatric Obesity Center, according to the national recommendations for adolescents (Plan National Nutrition Santé) depending on their age range and gender (12‐15 years, from 40 to 50 kcal/kg/d). The mean daily composition of macronutrients was 35% lipids, 55% carbohydrates, and 15% proteins (not exceed 0.9 g/kg/d). Participants did not receive any protein supplementation (as protein did not exceed 0.9 g/kg/d, which conforms with recommendations for their age range.

### 2.4 Maximal incremental exercise test

Each subject performed an incremental exercise test on a traditional CON ergometer. After a few minutes at a steady state (resting heart rate: 70.9 ± 8.2 bpm), the initial power was set at 30 W for 3 minutes, followed by 15 W increments every minute until exhaustion (pedal cadence was kept constant at 60‐70 revolutions per minute). The adolescents were strongly encouraged by the experimenters throughout the test to perform at maximal effort. The maximal exercise test was defined by at least two of the following criteria: heart rate > 90% of the theoretical maximum heart rate and respiratory exchange ratio (RER = $\dot{V}{CO}_{2}$/$\dot{V}O_{2}$) >1.1; $\dot{V}O_{2}$ plateau. Cardiac electrical activity was monitored continuously with heart rate telemetry (Ultima Series™, Saint Paul, MN) and combined with breath‐by‐breath gas exchange measurement (BreezeSuite Software, Saint Paul, MN) to determine $\dot{V}O_{2}$ and CO_2_ production ($\dot{V}{CO}_{2}$). $\dot{V}O_{2peak}$ was then defined as the average of the last 30 seconds of exercise before exhaustion at the maximal power output (*P*_max_).

### 2.5 Training

The training program consisted of three phases. Phase 1 involved 2 weeks of habituation (ie, progressive increase in exercise intensity and session length) in order to protect subjects from DOMS.^7^ During the first sessions, a load corresponding to 20% $\dot{V}O_{2peak}$ was imposed, with exercise duration gradually increased by 10‐minute increments up to 30 minutes. Once the exercise duration reached 30 minutes, the exercise intensity ramped up progressively by 10% until achieving 50% $\dot{V}O_{2peak}$. Phase 2 consisted of 45‐minute sessions with a 10‐minute warm‐up on CON cycle ergometers at 30% $\dot{V}O_{2peak}$, then 30 minutes ECC or CON cycling at 50% $\dot{V}O_{2peak}$, and a 5‐minute cool down. Phase 3 consisted of 45‐minute sessions with a 10‐minute warm‐up on CON ergocycles at 30% $\dot{V}O_{2peak}$, 30 minutes ECC or CON cycling at 70% $\dot{V}O_{2peak}$, and a 5‐minute cool down. Patients were asked for a rating of their perceived exertion (RPE) during each exercise.^28^ During the whole 12‐week training, the duration of the session and loads was not increased if participants suffered from DOMS, as indicated by scores >3 on a visual analogic scale (0‐10 scale) or when the rating of the perceived exertion (RPE) of the session was >13 according to BORG (6‐20 scale).

Eccentric cycling was conducted using commercial ECC motor‐driven ergometers (Cyclus2 Eccentric Recumbent; RBM elektronik‐automation; MSE Medical, Duttlenheim, France). The pedals were driven by a motor in a backward rotation and subjects had to try to slow the rotary motion by applying force, resulting in ECC muscle contractions of the extensor muscles. The CON group trained on CON ergometers (Optibike Med 600; MSE Medical). The ECC and CON ergometers both had adjustable recumbent seats. The same stance, positions, and angles between the trunk and legs during exercises were fixed in both training groups. Both revolutions per minute (fixed between 55 and 65) and load (power output, W) were controlled in both training groups. For CON exercises, power outputs were first evaluated from the initial maximal incremental tests. For ECC cycling, power outputs were calculated using a 1:3 ratio from the CON power outputs.^5^ In both the ECC and CON groups, power outputs were adjusted at the beginning of Phase 2 and Phase 3 using breath‐by‐breath gas exchange measurement for each adolescent, to ensure that the exercise intensity corresponded to the expected percentage of $\dot{V}O_{2peak}$, as described in the previous subsection (50% $\dot{V}O_{2peak}$ and 70% $\dot{V}O_{2peak}$, respectively). The adherence rate to exercise sessions was 100% in both groups during the whole 12 weeks of training, as any missed sessions were systematically caught up.

### 2.6 Anthropometric measurements and body composition

Weight and height were measured to an accuracy of 0.1 kg and 0.5 cm. Waist and hip circumference were measured by means of a flexible tape measure. Waist circumference was assessed between the last ribs and iliac crest. Body composition was evaluated using dual‐energy X‐ray absorptiometry (DXA) by a trained blinded technician (DXA; Hologic QDR‐4500A, Hologic, Bedford, MA, USA). Data were analyzed using the Hologic QDR Software for Windows (Version 12.6) (Hologic, Bedford, MA, USA), which integrates whole‐body measurement and standard body regions, such as trunk, arms, and legs, delineated by specific anatomical landmarks. From this, the FM percentage per DXA was calculated. Finally, three measurements of body composition were available for statistical analysis: on admission to the medical center (3 months before the beginning of the intervention study) and before and after ECC or CON training. The DXA was realized 3 days after the last training session, in a resting state on the morning, at least 3 hours apart from the last energy ingestion. The DXA technic has been found highly accurate for the measurement of (a) whole‐body fat, intra‐class correlation (ICC) = 0.999, coefficient of variation (CV) = 2.3%; (b) fat mass, ICC = 0.998, CV = 1.6%; and (c) lean mass, ICC = 0.995, CV = 0.3% and is highly reproducible.^29^

### 2.7 Strength tests

The isokinetic and isometric torques of the thigh from the dominant limb were tested by using a dynamometer chair (Humac R/Norm™, Stoughton, MA). Participants were comfortably seated, with the hip joint at 90° (0°= full extension). The distal shin pad of the dynamometer was attached 2‐3 cm proximally to the lateral malleolus with a strap. Straps were applied across the chest, pelvis, and mid‐thigh to minimize extraneous body movements. The alignment between the dynamometer rotational axis and knee joint rotation axis (lateral femoral epicondyle) was checked at the beginning of each session. Gravity effect torque was used to correct torque measurements. Subjects warmed up by performing 20 submaximal CON and ECC contractions with an angular velocity of 180°/s. Participants were likewise asked to complete three submaximal practice repetitions prior to each test series. For the isokinetic trials, the range of motion was 70° (from 80° to 10° of knee flexion). The CON measurements involved three maximal knee extensions and flexions performed at an angular velocity of 60°/s. Eccentric measurements consisted of three maximal contractions at an angular velocity of 30°/s. Participants were encouraged to push/pull as hard and as fast as possible and complete the full motion range. For the isometric trials, the knee joint was fixed at an angle of 45° of flexion. Three isometric knee extensions were performed, with participants asked to produce their maximal force as fast as possible and maintain the contractions for 4‐5 seconds. The best peak torque values of each trial were recorded.

### 2.8 Blood samples

Glycemia, insulinemia, and plasma levels of total cholesterol, LDL cholesterol, HDL cholesterol, and triglycerides were measured in venous blood between 08:00 and 09:00 am after overnight fasting before and after the training program (at least 48 hours after the last exercise). LDL cholesterol was computed using Friedewald's formula (LDL cholesterol [mmol/L] = cholesterol total [mmol/L] − HDL cholesterol [mmol/L] − triglyceride × 0.5 [mmol/L]). Insulin resistance was expressed using the homeostasis model assessment of insulin resistance index (HOMA‐IR), ([HOMA‐IR] = glycemia [mmol/L] × insulinemia [mUI/L]/22.5).^30^

### 2.9 Statistical analysis

The sample size estimation was based on previous works reported in the literature, involving 10‐15 subjects per group, particularly following Mueller et al^17^ methods describing a standard deviation of the change in total body fat of approximately 1.2%. Thus, to highlight a minimal difference of 1.7% between the CON and ECC groups, 11 subjects per group were needed for a two‐sided Type I error at 5% and a statistical power of 90%. The inclusion of 20 subjects per group was initially proposed to take into account the potential patients lost to follow‐up following 3 months of training. Yet given that almost all included patients completed the protocol (only one adolescent out of 24 dropped out, the adherence rate to exercise sessions was 100% for all the other participants) and no subject was excluded due to missing data, we recruited the number of subjects fixed by the sample size estimation proposed above. Randomization was performed using random size blocks (coin toss procedure). Statistical analyses were performed using Stata software, Version 13 (StataCorp, College Station, TX). The tests were two‐sided, with a Type I error set at *α* = 0.05. Continuous data were expressed as mean ± standard deviation (SD) or median [interquartile range] according to statistical distribution (assumption of normality assessed by using the Shapiro‐Wilk test). Random‐effects models for correlated data were created to measure time and group effects and their time x‐group interaction*,* taking into account inter‐ and intra‐patient variability (subject as random effect). The normality of residuals from these models was studied using the Shapiro‐Wilk test. When appropriate, a logarithmic transformation was proposed to achieve the normality of dependent outcome. Then, two multivariable analyses were performed after adjusting for covariates fixed according to univariate results and clinical relevance (age, gender, FM, or BMI variations between admission to the medical center and recruitment to the study) in order to compare the training group evolutions independently of the anterior modification of body composition or weight loss. We voluntarily chose to present the results with the first model, adjusted for age, gender, and FM variations, yet it should be noted that the results considered with the second model, adjusted for age, gender, and BMI variations, were entirely comparable in terms of statistical significance for all parameters analyzed. Results were expressed using Hedges's effect sizes and regression coefficients with 95% confidence intervals. Concerning non‐repeated measures, quantitative variables were compared between groups by means of Student's *t* test or the Mann‐Whitney test when the assumptions of *t* test were not met (normality and homoscedasticity analyzed using the Fisher‐Snedecor test). Categorical parameters were compared between groups using chi‐squared or Fisher's exact tests. The relationships between quantitative variables were studied while estimating correlation coefficients (Pearson's or Spearman's, according to statistical distribution with the Šidák Type I error correction due to multiple comparisons).

## 3 RESULTS

In total, 23 adolescents completed the study. One adolescent in the ECC group dropped out for family reasons, leaving n = 11 (six females and five males) in the ECC group and n = 12 in the CON group (six females and six males). The mean adolescent age was 13.3 *±* 1.2 years in the CON group and 13.6 ± 1.3 years in the ECC group (*P* = 0.49).

### 3.1 Intensities of training

During Phase 2 of training, $\dot{V}O_{2}$ was 1111 ± 263 mL/min (52.4% ± 6.1% of $\dot{V}O_{2peak}$) in the CON group and 1158 ± 281 mL/min (51.6% ± 4.3% of $\dot{V}O_{2peak}$) in the ECC group (*P* = 0.68). During Phase 3 of training, $\dot{V}O_{2}$ was 1517 ± 341 mL/min (69.4% ± 2.7% of $\dot{V}O_{2peak}$) in the CON group and 1597 ± 380 mL/min (69.2% ± 5.6% of $\dot{V}O_{2peak}$) in the ECC group (*P* = 0.60). Power output was nearly 3.7 times higher in the ECC group (59.2 ± 22.3 W vs 216.3 ± 70.2 W during Phase 2; 82.5 ± 36 W vs 313.3 ± 94.9 W during Phase 3, *P* < 0.001), with the same oxygen uptake and ventilation in the two groups.

### 3.2 Anthropometric measurements and body composition

**Figure 2:**
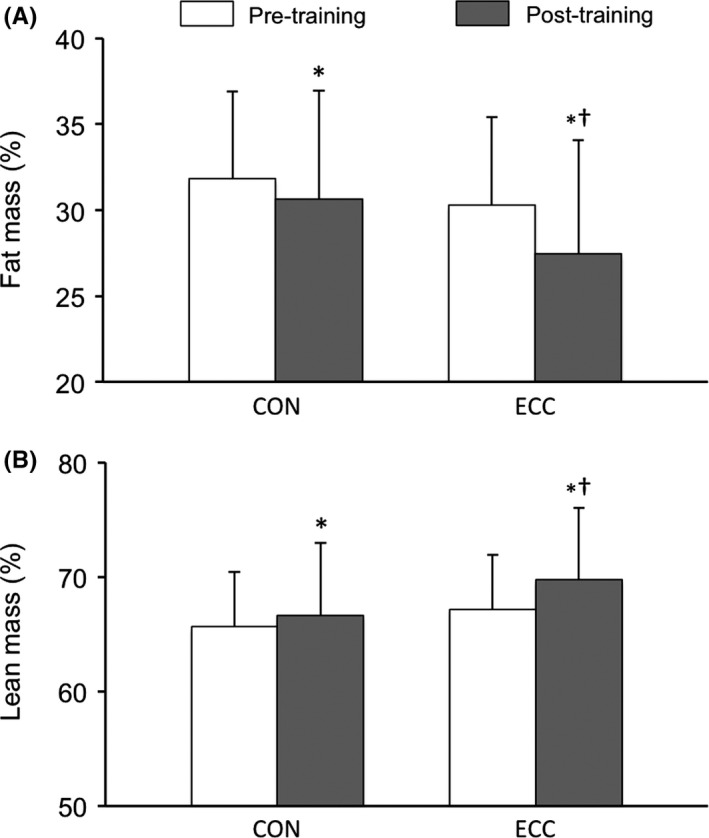
Comparison of (A) whole‐body fat and (B) whole‐body lean mass percentages before and after the 12‐wk cycling program for the concentric (CON) and the eccentric (ECC) groups in obese adolescents (n = 23; mean ± SD). Training effect *P <0.05; time *x*‐group interaction ^†^ *P* <0.05. Whole‐body fat mass percentage: ES 0.67 [−0.15 to 1.48]; CR 1.05 [0.06‐2.0] Whole‐body lean mass percentage: ES 0.69 [−0.13 to 1.50]; RC 1.00 [0.04‐1.95]. ES, effect size from univariate analysis; RG, regression coefficient from multivariate analysis

Concerning the primary outcome, whole‐body FM percentage significantly decreased after training compared to pretraining in both CON (−4.2% ± 4.9%) and ECC (−10.0% ± 9.2%) groups, with a significantly greater decrease in the ECC group when compared to the CON group (group × time interaction, *P* <0.05; Figure 2). BMI significantly decreased after training compared to pretraining in CON (−5.4% ± 2.8%) and ECC (−5.8% ± 4.9%) groups, without any significant difference between group × time interaction (*P* = 0.859). Whole‐body LM percentage significantly increased after training compared to pretraining in the CON (1.5% ± 2.4%) and ECC (3.8% ± 2.9%) groups, with a significantly greater increase in the ECC group than the CON group (group × time interaction, *P* <0.05; Figure 2). Absolute whole‐body LM did not significantly change after training compared to pretraining in the ECC group (−1.0 ± 4.3), while it decreased in the CON (−1.8 ± 3.2) group (*P* < 0.05). Leg FM decreased (−6.5% ± 5.4%, *P* <0.05) and leg LM increased (3.0% ± 2.7%) significantly after training compared to pretraining only in the ECC group (training effect, *P* <0.05). Trunk FM significantly decreased after training compared to pretraining in CON (−10.7% ± 9.5%) and ECC (−13.8% ± 12.8%) groups, without any significant difference between group × time interaction (*P* = 0.179). Anthropometrics and body composition characteristics are presented in Table 1.

**Table 1:** Anthropometric measurements and body composition parameters before and after the 12‐wk cycling program for the concentric (CON) and the eccentric (ECC) groups in obese adolescents (n = 23; mean ± SD)

	CON (n = 12)		ECC (n = 11)		*P*‐value	ES [95% CI]	RC [95% CI]
	Pre	Post	Pre	Post			
Weight (kg)	76.6 ± 12.1	74.1 ± 11.5***	82.8 ± 17.4	78.8 ± 16.0***	0.739	0.35 [−0.45 to 1.14]	0.25 [−1.24 to 1.75]
Height (cm)	159.6 ± 7.7	161.5 ± 7.5**	163.4 ± 7.2	164.5 ± 7.6**	0.849	0.45 [−0.35 to 1.25]	0.001 [−0.007 to 0.008]
BMI (kg/m^2^)	30.0 ± 3.5	28.4 ± 3.7***	30.8 ± 4.9	29.0 ± 4.5***	0.859	0.10 [−0.69 to 0.89]	0.05 [−0.49 to 0.59]
Z‐BMI (kg/m^2^)	2.01 ± 0.3	1.65 ± 0.5***	2.03 ± 0.4	1.7 ± 0.5***	0.849	−0.14 [−0.93 to 0.65]	−0.01 [−0.11 to 0.10]
BMI (percentile)	97.2 ± 2.2	93.8 ± 6.8*	96.8 ± 3.6	93.4 ± 6.7*	0.919	−0.14 [−0.93 to 0.65]	0.12 [−2.20 to 2.44]
Waist circumference (cm)	80.8 ± 5.7	81.8 ± 6.3	85.3 ± 9.1	83.5 ± 9.2*	0.046†	0.85 [0.001 to 1.69]	1.25 [0.02 to 2.46]
Hip circumference (cm)	103.3 ± 9.4	103.8 ± 9.1	105.0 ± 10.9	102.0 ± 9.2*	0.132	0.51 [−0.32 to 1.32]	0.95 [−0.72 to 2.62]
Whole‐body lean mass (kg)	50.3 ± 8.7	49.3 ± 8.5*	55.2 ± 10.3	54.6 ± 10.6	0.136	−0.18 [−0.96 to 0.61]	0.67 [−0.21 to 1.55]
Whole‐body fat mass (kg)	24.4 ± 5.7	22.8 ± 6.4**	25.5 ± 8.3	22.1 ± 8.2***	0.075	0.59 [−0.22 to 1.39]	0.95 [−0.19 to 2.09]
Leg mass (kg)	30.0 ± 6.1	29.4 ± 5.9	30.8 ± 7.7	30.1 ± 7.7	0.771	0.05 [−0.78 to 0.79]	0.11 [−0.62 to 0.81]
Leg lean mass (kg)	18.4 ± 3.8	17.8 ± 3.9**	19.7 ± 4.5	19.9 ± 4.5*	0.026†	0.82 [0.01 to 1.64]	0.51 [0.06 to 0.96]
Leg lean mass (%)	61.5 ± 5.8	60.7 ± 7.1	64.5 ± 5.5	66.4± 6.5*	0.009†	1.07 [0.21 to 1.92)	1.39 [0.34 to 2.43]
Leg fat mass (kg)	10.8 ± 3.3	10.8 ± 3.6	10.2 ± 3.7	9.3 ± 3.5*	0.034†	0.66 [−0.16 to 1.46]	0.47 [0.03 to 0.90]
Leg fat mass (%)	35.7 ± 6.2	36.4 ± 7.5	32.6 ± 5.8	30.6 ± 6.8*	0.010†	0.95 [0.10 to 1.78]	1.43 [0.34 to 2.53]
Trunk mass (kg)	38.1 ± 7.1	35.7± 7.2***	34.21 ± 5.0	32.6 ± 4.6***	0.800	0.32 [−0.47 to 1.11]	0.09 [−0.63 to 0.81]
Trunk lean mass (kg)	26.6 ± 4.3	26.2 ± 4.8	24.1 ± 4.0	23.8 ± 3.6	0.225	0.19 [−0.60 to 0.97]	0.25 [−0.16 to 0.66]
Trunk lean mass (%)	70.4 ± 5.2	73.9 ± 6.9***	70.4 ± 4.8	73.3 ± 5.8***	0.197	−0.21 [−0.99 to 0.58]	0.75 [−0.39 to 1.90]
Trunk fat mass (kg)	10.9 ± 3.5	9.0 ± 3.8***	9.60 ± 2.17	8.29 ± 2.6***	0.187	0.31 [−0.48 to 1.10]	0.36 [−0.18 to 0.91]
Trunk fat mass (%)	28.1 ± 5.4	24.5 ± 7.1***	28.1 ± 5.0	25.3 ± 6.0***	0.179	0.26 [−0.53 to 1.05]	0.79 [−0.36 to 1.94]

BMI, body mass index; CON, concentric cycling; ECC, eccentric cycling; ES, effect size from univariate analysis; RC, regression coefficient from multivariate analysis.

Training effect:

* *P* <0.05,

** *P* <0.01,

*** *P* <0.001.

† *P* <0.05: time *x*‐group interaction

### 3.3 Maximal incremental exercise tests, strength tests, and blood samples

**Figure 3:**
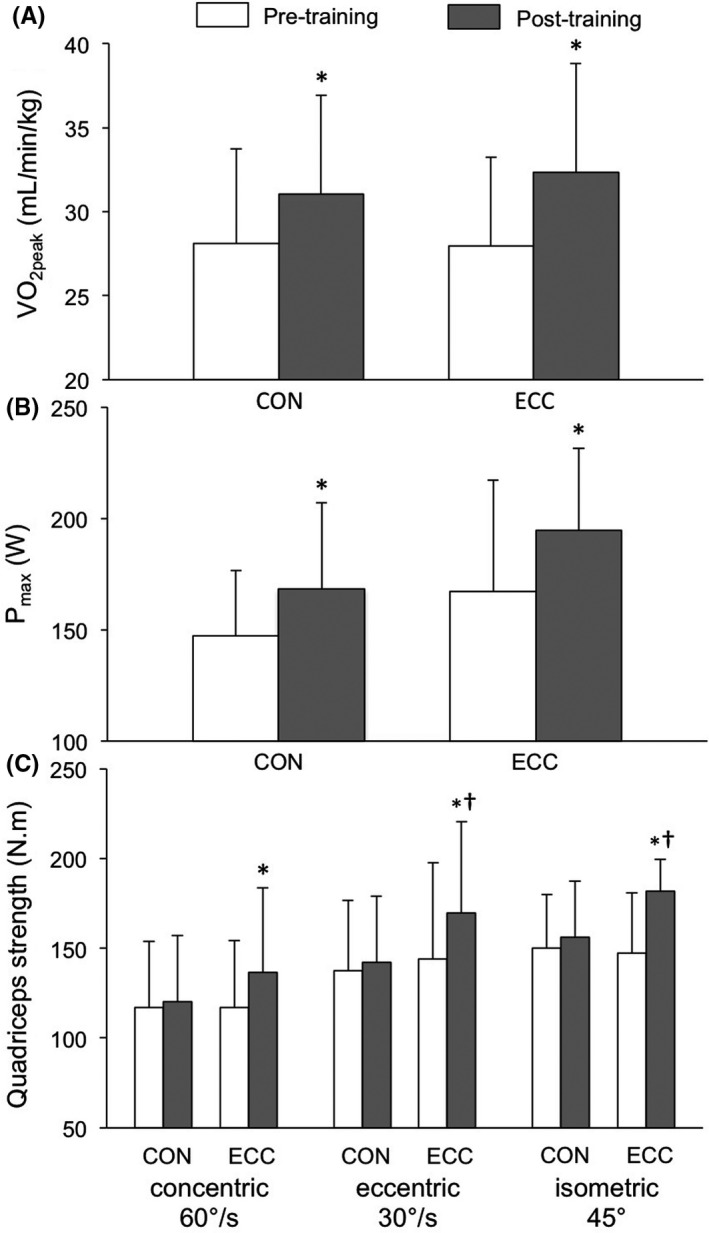
Comparison of (A) maximal oxygen consumption ($\dot{V}O_{2peak}$), (B) maximal power output (*P*_max_), and (C) quadriceps strength before and after the 12‐wk cycling program for the concentric (CON) and the eccentric (ECC) groups in obese adolescents (n = 23; mean ± SD). Training effect *P <0.05; time *x*‐group interaction ^†^ *P* <0.05. $\dot{V}O_{2peak}$: ES: 0.27 [−0.53 to 1.06]; RC: 41.9 [−53.0 to 136.8]; *P*_max_: ES: 0.44 [−0.37 to 1.23]; RC: 2.21 [−5.10 to 9.51]; isokinetic CON strength: ES: 0.44 [−0.37 to 1.23]; RC: 9.43 [−1.27 to 20.1]; isokinetic ECC strength: ES: 0.72 [−0.10 to 1.53]; RC: 10.5 [−1.17 to 22.2]; isometric strength: ES: 0.82 [0.05‐1.59]; RC: 15.4 [1.03‐29.8]. ES, effect size from univariate analysis; RC, regression coefficient from multivariate analysis

Figure 3 illustrates the main functional results of the maximal incremental tests and strength tests. $\dot{V}O_{2peak}$ increased in both training groups after training compared to pretraining in the ECC (16.6% ± 10.4%) and the CON (10.9% ± 9.8%) group (training effect, *P* < 0.05), with no significant difference in group × time interaction (*P* = 0.387). Quadriceps strength significantly increased in the ECC group only after training compared to pretraining for the concentric peak torque (16.2% ± 17.3%), the eccentric peak torque (21.3% ± 22.3%), and the isometric peak torque (28.3% ± 26.7%; training effect, *P* < 0.05). These increases were significantly greater in the ECC group compared to the CON group for eccentric peak torque (group × time interaction, *P* <0.05) and isometric peak torque (group × time interaction, *P* <0.05). Concentric, eccentric, and isometric peak torques indexed with leg LM significantly increased after training compared to pretraining in the ECC group (training effect, *P* < 0.01). There was no significant correlation between the increase in leg LM after training compared to pretraining and that of strength or $\dot{V}O_{2peak}$ (data not shown).

The blood sample results are provided in Table 2. The decrease in HOMA‐IR was significant after training compared to pretraining only in the ECC group (−19.9% ± 24.4%, *P* <0.05) due to a significant decrease in both insulinemia (−0.17% ± 0.20%, *P* < 0.05) and glycemia (−0.05% ± 0.06%, *P* < 0.05).

**Table 2:** Blood samplings parameters before and after the 12‐wk cycling program for the concentric (CON) and the eccentric (ECC) groups of obese adolescents (n = 23; mean ± SD)

	CON (n = 12)		ECC (n = 11)		*P*‐value	ES [95% CI]	RC [95% CI]
	Pre	Post	Pre	Post			
Insulinemia (mU/L)	19.23 ± 7.59	17.92 ± 8.34	23.24 ± 9.14	19.70 ± 9.70*	0.749	0.64 [−0.24 to 1.50]	0.42 [−2.15 to 2.99]
Glycemia (mmol/L)	4.66 ± 0.27	4.45 ± 0.35*	4.78 ± 0.21	4.55 ± 0.31*	0.968	0.04 [−0.88 to 0.80]	0.003 [−0.14 to 0.15]
HOMA‐IR	4.02 ± 1.71	3.54 ± 1.69	4.95 ± 1.85	4.03 ± 2.10*	0.762	0.53 [−0.34 to 1.39]	0.10 [−0.53 to 0.72]
Total cholesterol (mmol/L)	4.22 ± 0.77	4.11 ± 0.86	3.81 ± 0.63	3.71 ± 0.67	0.443	0.01 [−0.83 to 0.85]	0.08 [−0.12 to 0.28]
LDL cholesterol (mmol/L)	2.43 ± 0.67	2.26 ± 0.72	2.21 ± 0.44	2.09 ± 0.60	0.141	−0.26 [−1.10 to 0.59]	0.13 [−0.04 to 0.30]
HDL cholesterol (mmol/L)	1.36 ± 0.39	1.42 ± 0.32	1.20 ± 0.27	1.27 ± 0.28	0.276	0.53 [−0.34 to 1.38]]	0.03 [−0.02 to 0.09]
Triglycerides (mmol/L)	0.88 ± 0.28	0.87 ± 0.22	0.92 ± 0.27	0.75 ± 0.27	0.144	0.80 [−0.09 to 1.7]	0.11 [−0.04 to 0.25]

CON, concentric cycling; ECC, eccentric cycling; ES, effect size from univariate analysis; HOMA‐IR, homeostasis model assessment of insulin resistance index; RC, regression coefficient from multivariate analysis.

* *P* <0.05: Training effect.

### 3.4 Rate of perceived exertion

The average values of RPE were not significantly different between CON and ECC groups during Phase 1 (9.1 ± 2.4 vs 10.4 ± 1.8, *P* = 0.15), Phase 2 (9.6 ± 2.6 vs 11.2 ± 1.4, *P* = 0.08), and Phase 3 (10.6 ± 2.7 vs 11.5 ± 2.2, *P* = 0.39) of training.

## 4 DISCUSSION

This study sought to compare the effects of a 12‐week ECC vs CON cycling training on body composition, aerobic capacities, quadriceps strength, insulin resistance, and blood lipid profile in adolescents with obesity. To the best of our knowledge, this is the first study to analyze the effects of ECC and CON cycling training, performed at the same metabolic oxygen consumption level (similar exercise VO_2_), on body composition. According to our results, both CON and ECC interventions improve body composition and aerobic capacity. The improvements observed in our study after a 12‐week CON cycling training on whole‐body fat mass percentage (−4.2%), BMI (−5.4%), and VO_2peak_ (10.9%) are fully in line with the literature, which has extensively demonstrated its beneficial impact in obese population.^2^, ^3^, ^31^, ^32^ However, these improvements had greater magnitude after oxygen consumption‐matched ECC cycling training, in particular, greater whole‐body fat and lean mass improvements, with additional effects on quadriceps strength and insulin resistance.

The decreases in whole‐body FM (−10%) and leg FM percentages (−6.5%) observed following ECC cycling training in our study are concordant with that observed in previous studies, though these studies conducted the different training sessions at varying metabolic rates and mechanical powers, as well as using different moderate load ECC training modalities. Mueller et al^17^ compared the effects of a 12‐week training program on elderly sarcopenic patients, separated into one moderate load ECC group using a motorized ECC ergometer, one conventional resistance training group, and a control group. They demonstrated that only the ECC group exhibited a significant reduction in whole‐body FM (−5%) and thigh fat content (−6.9%), measured by DXA. Concerning muscle thigh fat infiltrations, which negatively correlate with insulin sensitivity, Marcus et al^19^ compared the impact of 16‐week aerobic training vs aerobic plus recumbent ECC stepping in adults with Type 2 diabetes mellitus, reporting that both interventions experienced a similar decrease in intramuscular fat, measured by magnetic resonance imaging (MRI). Thigh intramuscular adipose tissue has also been investigated using MRI in elderly adults but Jacobs et *al* failed to find any significant difference between their moderate load ECC training group (using ECC stepping ergometer) and the traditional training group after 12 weeks of intervention.^18^ Concerning trunk FM, we observed in our study a similar decrease in both CON (−10.7%) and ECC (−13.8%) groups. Although it is well known that CON training can decrease visceral adiposity, studies that measure the impact of ECC training on visceral FM are very limited. In a clinical trial carried out among postmenopausal women with impaired glucose tolerance, Marcus et al^14^ demonstrated that 12 weeks of moderate load ECC training (with motorized ECC ergometers) resulted in a decreased abdominal FM (whole‐body and segmental DXA measurements were not provided). It is of great interest as visceral adiposity has been established as a risk factor for the development of impaired glucose tolerance and cardiovascular diseases.^32^ Reduced waist circumference, and total and abdominal fat mass without loss of lean mass are thus excellent markers of reduced metabolic risk.^31^, ^32^

The greater whole‐body FM decrease observed in our ECC group could be partly explained by a physiological and metabolic hypothesis that ECC training impacts resting energy expenditure and metabolic substrates. It has previously been demonstrated that acute and chronic ECC exercises (based on ECC quadriceps lengthening contractions via isokinetic dynamometry) can induce a larger increase in post‐training resting energy expenditure compared to CON (based on CON quadriceps shortening contractions via isokinetic dynamometry) when performed using the same mechanical load. This could be primarily due to the enhanced muscle protein turnover, which arises because of inflammatory processes to repair EIMD and support muscle hypertrophy.^22^ Although the repeated bout effect seems to reduce the increase in postexercise resting energy expenditure (which is attenuated after bouts of ECC exercise),^22^ the multiple increase in intensity of exercise in our intervention (from 20% $\dot{V}O_{2peak}$ to 70% VO_2peak_) gives reason to think that the repairing processes, associated with enhanced postexercise energy expenditure, may continue. It has likewise been suggested that ECC training could impact metabolic substrate use, increasing the fat oxidation rate while reducing that of glucose.^22^ These explicative mechanisms seem to be emphasized in patients with overweight and obesity in comparison with lean subjects.^33^ The effect of ECC training on lipid oxidation, associated with a more oxidative metabolism, has been supported by several studies involving both human and mice models.^34^, ^35^

The increased whole‐body LM (+3.5%) and leg LM percentages (+3.0%) observed following moderate load ECC cycling in the present study are consistent with the well‐known ability of skeletal muscle to repair and regenerate in response to ECC exercise, leading to muscle hypertrophy.^20^ As hypertrophy is considered a strong determining factor for force production, and given that meta‐analyses have highlighted that ECC contractions improve muscle strength to a greater extent than CON contractions,^13^, ^20^ the greater gain in strength we observed after ECC training was expected. Since this strength gain did not significantly correlate with the gain in LM, and given that peak torques relative to leg LM are significantly higher after training in the ECC group, there could be factors other than LM at play, such as neuronal factors (increases in motor unit discharge rates, improvements in agonist muscle voluntary activations via disinhibition of excitatory input to spinal motor neurons, and changes in motor unit recruitments, along with morphological and architectural adaptations)^20^ The observed rises in quadriceps’ strength are even more important when considering the results of a recent meta‐analysis highlighting that lower limb performances are reduced in children with obesity compared to their lean peers, especially if strength is expressed in terms of body mass.^36^ Furthermore, when managing obesity, more attention should be paid to LM, as well as FM, measurements. Lean mass, unlike FM or BMI, is correlated in adults and adolescents with obesity to resting metabolic rate and energy intake, and could exert a determining effect on energy intake through satiation control.^37^ Moreover, in adults with obesity, LM depletion associated with weight loss could lead to a hyperphagic response and enhance the phenomenon of fat overshooting.^38^ The weight loss we observed in our patients, absent of any LM depletion after ECC cycling training, can thus be considered a strong positive effect for the long‐term management of obesity.

Otherwise, we found the aerobic capacities of our patients, measured by VO_2peak_, were improved in both groups (by 10% in the CON group and by 15% in the ECC group). Aerobic capacity measurements are now considered major independent predictive parameters of mortality in both adult and pediatric subjects with cardiovascular risk factors.^39^ In our study, as a consequence of matching metabolic load in the training groups, the similar VO_2peak_ gain between groups was considered an expected result.

Our results also demonstrate that moderate load ECC cycling training has significant positive beneficial effects on insulin resistance. The HOMA‐IR is a non‐invasive technique that remains closely correlated with the gold standard euglycemic hyperinsulinemic clamp technique.^30^ Contrary to acute ECC exercises, which increase insulin resistance, chronic ECC exercises have been reported to decrease insulin resistance.^22^, ^23^, ^40^ Paschalis et al^22^ reported that only 30 minutes of ECC exercise (based on ECC quadriceps lengthening contractions via isokinetic dynamometry) per week for 8 weeks was sufficient to increase insulin sensitivity. Decreased insulin resistance following moderate load ECC training was similarly supported by Drexel et al^23^ in their results of a chronic downhill hiking model. The advanced mechanisms supporting improvement in insulin sensitivity following ECC training in our study consist of decreased waist circumference, trunk fat mass, and increased lean mass. These mechanisms can be associated both with increased fat oxidation rate, which prevents the accumulation of fatty acid‐derived metabolites in skeletal muscle, as evidenced in our study by decreased percentage of leg fat mass and increased muscle glucose intake. Others have reported improvement in plasma triglyceride levels (which are able to impair insulin action).^22^ Concerning the lipid profile, our study failed to show any significant improvements. This could be partially explained by our relatively small sample size and the near‐normal baseline levels of lipid profile parameters observed at the beginning of our interventions.

Finally, our study demonstrates for the first time the tolerance and feasibility of moderate load ECC cycling protocols in a pediatric population. In adults, moderate‐intensity ECC cycling trainings had previously been shown to be well tolerated provided the initial progressive ramping time was respected.^7^ The mechanical load we imposed to obtain the same VO_2_ in both training groups was roughly 3.7 times higher in the ECC group. This ratio supports previous physiological studies. As for achieving the same mechanical power, the VO_2_ measured was three‐to‐four times lower during ECC cycling compared to CON cycling.^5^ Although higher average values of RPE were expected in the ECC group due to the higher mechanical load developed, the difference in RPE between the ECC and CON groups remained nonsignificant, indicating a good tolerance of the ECC modality in adolescents with obesity. This tolerance remains concordant with previous observations, with the same acute ECC muscle stimulus having been reported to demonstrate that children and adolescents had less pronounced and shorter muscle damages with less functional impairments than adults.^41^, ^42^ Accelerated speeds of recovery have also been highlighted in young people.^25^, ^26^ The hypothesis proposed to explain this greater tolerance to ECC training is a combination of the higher component of ECC movements in the habitual physical activity of children (hopping, climbing, jumping) compared to adults, the greater flexibility of muscle fibers leading to less overextension of sarcomeres, and the lower proportion of fast‐twitch fibers compared to adults, which are known to be more susceptible to damage.^26^, ^27^

The decision to enroll adolescents 3 months after their admission to the medical center could be criticized, as some results presented here (particularly weight and FM loss during training) may be underestimated in comparison with clinical trials carried out on patients in the first stage of their medical treatment. Carnier et al, for example, indicated that obese adolescent weight loss was approximately three times greater in the first 6 months of medical treatment than in the 6 months after.^2^ These results should encourage scientists and practitioners to focus their attention and efforts on developing new more effective programs to implement in the second phase of this weight loss process in order to optimize the benefits of long‐term weight loss interventions. Based on this literature and regarding the fact that it seems difficult to directly enroll inactive obese adolescents in specific trainings such as ECC that involves great muscle effort,^43^ we decided to focus our intervention on the participants’ second phase of weight loss. Significantly, our statistical analysis took into consideration changes in the decrease in FM (and BMI) between the admission to the medical center and enrollment in the study, demonstrating that ECC training is significantly superior to CON training in terms of reducing FM independently of any anterior modification of body composition (and weight loss). Thus, from a clinical point of view, our results appear even more promising as they prove that ECC cycling training is highly effective at the time when classic medical care proves usually less efficient.

It must be pointed out that adolescents were not following individually restrictive dietary interventions neither protein supplementations. They all received the same daily energy intake respecting the nutritional recommendations for their age and gender. The supervised nature of our clinical center guaranteed our control of their daily intake (number of calories and percentages of macronutrients), which were thus not different between groups.

Finally, it would have been of substantial interest to conduct follow‐up assessments to analyze the potential long‐term effects of the two interventions; however, this was not possible in this study because of practical reasons.

## 5 PERSPECTIVES

Although both ECC and CON cycling trainings are efficient in improving body composition, greater adiposity reduction and muscle mass increase are observed in response to ECC compared to CON training performed at the same metabolic rate in obese adolescents. The relevance of these results is even greater considering ECC training is more efficient in reducing FM and is independent of any anterior modification of body composition and weight loss (first phase of a weight loss program), and is observed during the second phase of the clinical intervention when traditional combined nutritional and physical activity programs appear to be less successful. Our study shows that ECC cycling training represents an optimal and appropriate modality of training to be recommended to maintain and prolong weight loss and decrease global, trunk, and segmental FM in adolescents with obesity, in association with well‐balanced food intake. Moreover, ECC cycling training is well tolerated and bears additional effects on muscle mass, muscle strength, and metabolic risk parameters such as insulin resistance. Additionally, it will be necessary in the future to compare the effectiveness of ECC and CON cycling programs performed in standardized experimental conditions of power output, the same mechanical power with a lower VO_2_ in the ECC group.

## 6 ETHICS APPROVAL

Ethical approval has been obtained from Ethics Committee of Strasbourg (East IV), France (IDRCB 2016‐A00043‐48). ClinicalTrials identifier: NCT02275455.

## CONFLICT OF INTEREST

All authors declare that there is no conflict of interest. The authors alone are responsible for the content and writing of the manuscript.

## References

[1] Daniels SR , Arnett DK , Eckel RH , et al. Overweight in children and adolescents: pathophysiology, consequences, prevention, and treatment. Circulation. 2005;111(15):1999‐2012. 15837955 10.1161/01.CIR.0000161369.71722.10

[2] Carnier J , de Mello MT , Ackel‐DElia C , et al. Aerobic training (AT) is more effective than aerobic plus resistance training (AT+RT) to improve anorexigenic/orexigenic factors in obese adolescents. Appetite. 2013;69:168‐173. 23764241 10.1016/j.appet.2013.05.018

[3] García‐Hermoso A , Ramírez‐Vélez R , Ramírez‐Campillo R , Peterson MD , Martínez‐Vizcaíno V . Concurrent aerobic plus resistance exercise versus aerobic exercise alone to improve health outcomes in paediatric obesity: a systematic review and meta‐analysis. Br J Sports Med. 2018;52(3):161‐166. 27986760 10.1136/bjsports-2016-096605

[4] Hoppeler H . Moderate load eccentric exercise; A distinct novel training modality. Front Physiol. 2016;7:483. 27899894 10.3389/fphys.2016.00483 PMC5110564

[5] Perrey S , Betik A , Candau R , Rouillon JD , Hughson RL . Comparison of oxygen uptake kinetics during concentric and eccentric cycle exercise. J Appl Physiol (1985). 2001;91(5):2135‐2142. 11641354 10.1152/jappl.2001.91.5.2135

[6] Peñailillo L , Blazevich AJ , Nosaka K . Factors contributing to lower metabolic demand of eccentric compared with concentric cycling. J Appl Physiol (1985). 2017;123(4):884‐893. 28663378 10.1152/japplphysiol.00536.2016

[7] LaStayo P , Marcus R , Dibble L , Frajacomo F , Lindstedt S . Eccentric exercise in rehabilitation: safety, feasibility, and application. J Appl Physiol (1985). 2014;116(11):1426‐1434. 23823152 10.1152/japplphysiol.00008.2013

[8] Tidball JG . Mechanisms of muscle injury, repair, and regeneration. Compr Physiol. 2011;1(4):2029‐2062. 23733696 10.1002/cphy.c100092

[9] Peñailillo L , Blazevich A , Numazawa H , Nosaka K . Metabolic and muscle damage profiles of concentric versus repeated eccentric cycling. Med Sci Sports Exerc. 2013;45(9):1773‐1781. 23475167 10.1249/MSS.0b013e31828f8a73

[10] McHugh MP . Recent advances in the understanding of the repeated bout effect: the protective effect against muscle damage from a single bout of eccentric exercise. Scand J Med Sci Sports. 2003;13(2):88‐97. 12641640 10.1034/j.1600-0838.2003.02477.x

[11] LaStayo P , Marcus R , Dibble L , Wong B , Pepper G . Eccentric versus traditional resistance exercise for older adult fallers in the community: a randomized trial within a multi‐component fall reduction program. BMC Geriatr. 2017;17(1):149. 28716003 10.1186/s12877-017-0539-8 PMC5513167

[12] LaStayo PC , Pierotti DJ , Pifer J , Hoppeler H , Lindstedt SL . Eccentric ergometry: increases in locomotor muscle size and strength at low training intensities. Am J Physiol Regul Integr Comp Physiol. 2000;278(5):R1282‐R1288. 10801298 10.1152/ajpregu.2000.278.5.R1282

[13] Roig M , O’Brien K , Kirk G , et al. The effects of eccentric versus concentric resistance training on muscle strength and mass in healthy adults: a systematic review with meta‐analysis. Br J Sports Med. 2009;43(8):556‐568. 18981046 10.1136/bjsm.2008.051417

[14] Marcus RL , Lastayo PC , Dibble LE , Hill L , McClain DA . Increased strength and physical performance with eccentric training in women with impaired glucose tolerance: a pilot study. J Womens Health. 2009;18(2):253‐260. 10.1089/jwh.2007.0669 PMC2945716 19183097

[15] Schoenfeld BJ , Grgic J , Ogborn D , Krieger JW . Strength and hypertrophy adaptations between low‐ vs. high‐load resistance training: a systematic review and meta‐analysis. J Strength Cond Res. 2017;31(12):3508‐3523. 28834797 10.1519/JSC.0000000000002200

[16] Franchi MV , Reeves ND , Narici MV . Skeletal muscle remodeling in response to eccentric vs. concentric loading: morphological, molecular, and metabolic adaptations. Front Physiol. 2017;8:447. 28725197 10.3389/fphys.2017.00447 PMC5495834

[17] Mueller M , Breil FA , Vogt M , et al. Different response to eccentric and concentric training in older men and women. Eur J Appl Physiol. 2009;107(2):145‐153. 19543908 10.1007/s00421-009-1108-4

[18] Jacobs JL , Marcus RL , Morrell G , LaStayo P . Resistance exercise with older fallers: its impact on intermuscular adipose tissue. BioMed Res Int. 2014;2014:398960. 24804220 10.1155/2014/398960 PMC3996328

[19] Marcus RL , Smith S , Morrell G , et al. Comparison of combined aerobic and high‐force eccentric resistance exercise with aerobic exercise only for people with type 2 diabetes mellitus. Phys Ther. 2008;88(11):1345‐1354. 18801851 10.2522/ptj.20080124 PMC2579905

[20] Douglas J , Pearson S , Ross A , McGuigan M . Chronic adaptations to eccentric training: a systematic review. Sports Med. 2016;47(5):917‐941. 10.1007/s40279-016-0628-4 27647157

[21] Peñailillo L , Blazevich A , Nosaka K . Energy expenditure and substrate oxidation during and after eccentric cycling. Eur J Appl Physiol. 2014;114(4):805‐814. 24390692 10.1007/s00421-013-2816-3

[22] Paschalis V , Nikolaidis MG , Theodorou AA , et al. A weekly bout of eccentric exercise is sufficient to induce health‐promoting effects. Med Sci Sports Exerc. 2011;43(1):64‐73. 20508540 10.1249/MSS.0b013e3181e91d90

[23] Drexel H , Saely CH , Langer P , et al. Metabolic and anti‐inflammatory benefits of eccentric endurance exercise—a pilot study. Eur J Clin Invest. 2008;38(4):218‐226. 18339002 10.1111/j.1365-2362.2008.01937.x

[24] Croix M . Advances in paediatric strength assessment: changing our perspective on strength development. J Sports Sci Med. 2007;6(3):292‐304. 24149415 PMC3787279

[25] Gorianovas G , Skurvydas A , Streckis V , Brazaitis M , Kamandulis S , McHugh MP . Repeated bout effect was more expressed in young adult males than in elderly males and boys. Biomed Res Int. 2013;2013:218970. 23484095 10.1155/2013/218970 PMC3581300

[26] Marginson V , Rowlands AV , Gleeson NP , Eston RG . Comparison of the symptoms of exercise‐induced muscle damage after an initial and repeated bout of plyometric exercise in men and boys. J Appl Physiol (1985). 2005;99(3):1174‐1181. 15817716 10.1152/japplphysiol.01193.2004

[27] Lin M‐J , Nosaka K , Ho C‐C , et al. Influence of maturation status on eccentric exercise‐induced muscle damage and the repeated bout effect in females. Front Physiol. 2017;8:1118. 29354073 10.3389/fphys.2017.01118 PMC5760894

[28] Scherr J , Wolfarth B , Christle JW , Pressler A , Wagenpfeil S , Halle M . Associations between Borg’s rating of perceived exertion and physiological measures of exercise intensity. Eur J Appl Physiol. 2013;113(1):147‐155. 22615009 10.1007/s00421-012-2421-x

[29] Moreira OC , de Oliveira C , De Paz JA . Dual energy X‐ray absorptiometry (DXA) reliability and intraobserver reproducibility for segmental body composition measuring. Nutr Hosp. 2018;35:340‐345. 29756967 10.20960/nh.1295

[30] Henderson M , Rabasa‐Lhoret R , Bastard J‐P , et al. Measuring insulin sensitivity in youth: How do the different indices compare with the gold‐standard method? Diabetes Metab. 2011;37(1):72‐78. 21126900 10.1016/j.diabet.2010.06.008

[31] LeMura LM , Maziekas MT . Factors that alter body fat, body mass, and fat‐free mass in pediatric obesity. Med Sci Sports Exerc. 2002;34(3):487‐496. 11880814 10.1097/00005768-200203000-00016

[32] Kay SJ , Fiatarone Singh MA . The influence of physical activity on abdominal fat: a systematic review of the literature. Obes Rev. 2006;7(2):183‐200. 16629874 10.1111/j.1467-789X.2006.00250.x

[33] Paschalis V , Nikolaidis MG , Giakas G , et al. Beneficial changes in energy expenditure and lipid profile after eccentric exercise in overweight and lean women. Scand J Med Sci Sports. 2010;20(1):e103–e111. 19422638 10.1111/j.1600-0838.2009.00920.x

[34] Hody S , Leprince P , Sergeant K , et al. Human muscle proteome modifications after acute or repeated eccentric exercises. Med Sci Sports Exerc. 2011;43(12):2281‐2296. 21606878 10.1249/MSS.0b013e318222edf3

[35] Hody S , Lacrosse Z , Leprince P , Collodoro M , Croisier J‐L , Rogister B . Effects of eccentrically and concentrically biased training on mouse muscle phenotype. Med Sci Sports Exerc. 2013;45(8):1460‐1468. 23439418 10.1249/MSS.0b013e3182894a33

[36] Thivel D , Ring‐Dimitriou S , Weghuber D , Frelut M‐L , O’Malley G . Muscle strength and fitness in pediatric obesity: a systematic review from the European Childhood Obesity Group. Obes Facts. 2016;9(1):52‐63. 26901423 10.1159/000443687 PMC5644904

[37] Blundell JE , Caudwell P , Gibbons C , et al. Body composition and appetite: fat‐free mass (but not fat mass or BMI) is positively associated with self‐determined meal size and daily energy intake in humans. Br J Nutr. 2012;107(3):445‐449. 21733267 10.1017/S0007114511003138

[38] Dulloo AG , Jacquet J , Montani J‐P , Schutz Y . How dieting makes the lean fatter: from a perspective of body composition autoregulation through adipostats and proteinstats awaiting discovery. Obes Rev. 2015;16(Suppl 1):25‐35. 25614201 10.1111/obr.12253

[39] Ruiz JR , Castro‐Piñero J , Artero EG , et al. Predictive validity of health‐related fitness in youth: a systematic review. Br J Sports Med. 2009;43(12):909‐923. 19158130 10.1136/bjsm.2008.056499

[40] Philippe M , Gatterer H , Eder EM , et al. The effects of 3 weeks of uphill and downhill walking on blood lipids and glucose metabolism in pre‐diabetic men: a pilot study. J Sports Sci Med. 2017;16(1):35‐43. 28344449 PMC5358029

[41] Chen TC , Chen H‐L , Liu Y‐C , Nosaka K . Eccentric exercise‐induced muscle damage of pre‐adolescent and adolescent boys in comparison to young men. Eur J Appl Physiol. 2014;114(6):1183‐1195. 24563093 10.1007/s00421-014-2848-3

[42] Deli CK , Fatouros IG , Paschalis V , et al. A comparison of exercise‐induced muscle damage following maximal eccentric contractions in men and boys. Pediatr Exerc Sci. 2017;29:316‐325. 28165870 10.1123/pes.2016-0185

[43] O’Malley G , Ring‐Dimitriou S , Nowicka P , et al. Physical activity and physical fitness in pediatric obesity: what are the first steps for clinicians? Expert conclusion from the 2016 ECOG workshop. Int J Exerc Sci. 2017;10(4):487‐496. 28674594 10.70252/YFYO2813 PMC5466409

